# Wheat’s Up with CRISPR-Cas—Current Advances, Obstacles and Perspectives

**DOI:** 10.3390/ijms27135860

**Published:** 2026-06-29

**Authors:** Monika Samoń, Mateusz Przyborowski

**Affiliations:** Plant Breeding and Acclimatization Institute–National Research Institute, Radzików, 05-870 Błonie, Poland

**Keywords:** genome editing, editing methods, wheat, plant transformation, CRISPR-Cas

## Abstract

The emergence of CRISPR-Cas editing systems—comprising clustered regularly interspaced short palindromic repeats and associated Cas proteins—marked a breakthrough in genetic engineering, owing to the simplicity, efficiency, and adaptability of the method. Despite continuous improvements and the incorporation of innovative discoveries to develop reliable, fine-tuned tools, the effective application of CRISPR-Cas technology in cereals remains challenging. This review provides a technically oriented overview of CRISPR-Cas-mediated genome editing in wheat (*Triticum aestivum* L.), one of the world’s fundamental crops. While focusing on established solutions and progressive methodological modifications, we also discuss pertinent topics, including plant genetic transformation, prospective innovations, and compliance considerations.

## 1. Introduction

Until recently, the introduction and conservation of desirable traits in cultivated crop species was a laborious process relying on selective breeding, cross-breeding, or random mutagenesis; nowadays, the genome editing techniques allow for quicker and more precise modifications. Moreover, due to advances in molecular research, it is possible to target traits with no obvious phenotypic representation. The main modern genome editing methods use sequence-specific nucleases to generate double-strand breaks (DSBs) in the target DNA. DSBs activate cellular repair mechanisms, mainly non-homologous end joining (NHEJ) and homology-directed repair (HDR) pathways. These processes can be employed to introduce desired modifications [1]. The error-prone NHEJ pathway can result in random, small indels at the break site, usually leading to gene knockouts, whereas the HDR pathway allows for precise point mutations, as well as insertions or replacements, provided the presence of a homologous DNA template [2].

The systems of clustered regularly interspaced short palindromic repeats and associated proteins (CRISPR-Cas) are considered to be a simple, versatile, cheap, and effective tool for genomic engineering; nevertheless, there are numerous factors affecting the outcome of its application [3,4]. In this review, we focus on describing hitherto advances in wheat (*Triticum aestivum* L.) genome engineering using the CRISPR-Cas system and related techniques. As one of the world’s most important cultivated plants, wheat is definitely an essential subject of research on improving crop plants, with enhancing the stress tolerance of elite cultivars being one of the most critical concerns [5,6,7]. However, due to the properties of its genome (i.e., hexaploidy, size of approximately 17 GB, and 85% of repeated sequences), inducing effective modifications in this species, as well as transformation and regeneration, is regarded as difficult [7,8]. Considering the ongoing advances, we intend to summarize the current progress and outline future perspectives in this field. Although CRISPR–Cas systems have demonstrated remarkable potential for wheat genome engineering, further advances in editing efficiency, delivery, and plant regeneration are required before their full benefits can be translated into routine crop improvement.

## 2. Cas9 Nuclease—Characteristics and Sequence Optimization

The most widely used CRISPR gene editing system—CRISPR-Cas9—is based on the activity of the *Streptococcus pyogenes* Cas9 protein (SpCas9), an endonuclease of bacterial origin, which can generate blunt-ended DSBs in DNA. The selection of the cleavage site depends on the guide RNA (gRNA), which binds with Cas9 protein and directs the complex to the DNA sequence complementary to a spacer—a ~20-nucleotide fragment of gRNA. The sequence recognized by the spacer must be directly followed by a protospacer-adjacent motif (PAM) for the cleavage to occur [9,10]. The canonical PAM for native SpCas9 is NGG; other sequences, such as NAG and NGA, were reported to enable cleaving with lower efficiency [11]. Whereas natively Cas9 functionality requires two RNA components—CRISPR RNA (crRNA) and trans-activating CRISPR RNA (tracrRNA) for target recognition and protein activation, respectively—in biotechnological applications, these two molecules are replaced by a chimeric construct—single-guide RNA (sgRNA) [3,12].

The *Cas9* gene introduced to the host cells does not need to be rigidly conserved, and it has been shown that certain modifications can increase the performance of the technique or change the properties of the enzyme [13,14,15]. Specifically, inserting introns in the gene sequence has been shown to improve the efficiency of the system, presumably by increasing the protein expression [15]. Furthermore, the transgene sequence can be codon-optimized to better match the host’s expression apparatus, therefore mitigating the effect of codon usage bias [16]. Mutagenesis in wheat has been achieved with human, maize, rice, and wheat codon-optimized versions of *Cas9* [17,18,19]. Direct comparison of editing efficiency in wheat protoplasts revealed that *Cas9* with codon optimization for wheat outperformed sequences optimized for rice and human (with reported efficiency of 8%, 4.1%, and 3.6%, respectively) but was surpassed by the maize codon-optimized version (11%) [18]. This result was confirmed in transgenic wheat lines where the expression of maize codon-optimized *Cas9* was reported to be ~15 times higher than the wheat codon-optimized version [20]. Whereas some toolkits designed for gene editing in plants use the maize codon-optimized sequence [15], other authors chose the wheat codon-optimized *Cas9* for the expression in monocots [21,22]. Additionally, flanking Cas9 with nuclear localization signals (NLSs) directs the protein to the proper subcellular compartment, further increasing the editing efficiency [15,23]. The studies investigating the effects of different NLS designs reveal that the performance of the CRISPR technique can be affected by the position, number, and type of NLSs [24]. For example, it has been observed that Cas9 with histone H2B NLS at the C terminus outperformed the variants with Simian virus 40 (SV40) and nucleoplasmin NLSs in wheat protoplasts [22]. Moreover, placing NLSs at both termini results in higher editing efficiency than incorporating only one NLS [13].

## 3. Expression Cassette for the Nuclease and gRNA

Another essential factor affecting heterologous protein expression is the selection of transcriptional regulatory elements. One of the most commonly used sets, which proved to provide a high level of expression in monocots, is the maize ubiquitin promoter (ZmUbi) and *Agrobacterium tumefaciens* nopaline synthase (NOS) terminator [25,26]. Successful expression has also been reported with rice ubiquitin (OsUbi) and actin (OsAct) promoters, as well as *Sorghum bicolor* actin terminator, *Agrobacterium tumefaciens* octopine synthase terminator, and 35S-NOS double terminator [23,27,28,29,30]. The transcription of gRNA is typically driven by wheat RNA polymerase III promoters (TaU3 or TaU6) and uses five to seven thymidines as a transcription termination signal [31,32]. It has been shown that using species-derived promoters enhances editing efficiency in wheat [28]. Direct comparison in *Agrobacterium*-transformed wheat demonstrated that the TaU3 promoter outperformed both the TaU6 and OsU6a promoters, with reported mutation rates of 61.4%, 36.0%, and 21.6%, respectively [33]. Nevertheless, both wheat polymerase III promoters are incorporated into Cas-based genome editing toolkits and employed in multiplex constructs [15,34]. U3 and U6 promoters have a preference for the first transcribed nucleotide to be either A or G, respectively, which may limit their application. On the other hand, transcripts expressed from polymerase II promoters get processed, resulting in altered structure and reduced functionality [31]. Nonetheless, application of RNA polymerase II transcription regulatory elements—*Panicum virgatum* ubiquitin (PvUbi) promoter, Cestrum yellow leaf curling virus (CmYLCV) promoter, NOS terminator, and 35S terminator—for gRNA expression has been reported [21,35]. Since this system allows for producing longer transcripts, it is advantageous in the case of simultaneous expression of multiple gRNA sequences in multiplex editing. By flanking each individual gRNA with a separation signal, the unnecessary modifications can be removed, and the transcript of multiple gRNAs can be split into individual particles. The separation is performed by tRNA-processing enzymes, Csy4 protein, or ribozymes; out of the three methods, the Csy4-based approach was shown to provide the best efficiency [21]. Moreover, every one of these multi-gRNA designs outperforms the system where individual gRNA sequences are transcribed from separate polymerase III promoters [21]. The transcription of both the Cas nuclease and the gRNA(s) can also be driven by a single polymerase II promoter, resulting in a single transcription unit (STU) system, with individual elements separated by ribozyme cleavage sites [36,37]. The benefits of this approach include overcoming limitations associated with polymerase III promoters, reducing the construct size, and possible coordination/induction of expression [37]. An interesting approach to STUs is incorporating gRNA sequence as an intron of the Cas protein sequence [38]. To date, this system has been tested in rice protoplasts and has been demonstrated to be an efficient technique of multiplex genome editing, with its compact structure as an additional advantage; whether this method performs well in wheat is yet to be determined.

## 4. Balancing Efficiency and Specificity

Apart from designing a setup providing high expression of the CRISPR-Cas system components, numerous studies focus on methods to increase its working efficiency by modifying the conditions of the procedure. It has been reported that the frequency of Cas-induced mutations in wheat may be increased by heat treatment. Exposing immature embryos to a temperature of 37 °C for one day during the resting step was shown to increase the number of genome-edited wheat plants from 0.85% to 1.03% of embryos transformed with Cas9 [28]. Mutagenesis (mostly by increasing frequency and length of deletions) can also be enhanced by co-expressing an exonuclease along with a Cas endonuclease [39]. For example, it was demonstrated that human three prime repair exonuclease 2 (TREX2) improves the performance of the Cas9-based editing system in cereals. Earlier experiments showed that using constructs of Cas9, autocleavage peptide motif P2A, and TREX2 enhanced the editing frequency in barley and triticale protoplasts up to 2.5- and 15-fold, respectively [21,40]. The system’s effectiveness was also confirmed in transgenic wheat, although it was outperformed by a variant employing Cas9 and TREX2 fused by a 12-amino-acid linker [41]. A similar effect was observed for exonuclease T5 (T5exo) fused with Cas9 and Cas12a. Compared to constructs with a Cas nuclease only, T5exo-Cas9 had up to 4.3-fold higher editing efficiency in rice protoplasts and 1.07-fold increased mutagenesis frequency in transgenic rice plants; for T5exo-Cas12a, the reported enhancement was 1.47-fold and 1.75-fold, respectively [39]. Schreiber et al. used a fusion protein of Cas9 and 5′ exonucleases from the herpes virus (HSV exonucleases) to generate longer 3′ overhangs in the edited site, therefore increasing the frequency of HDR; this approach resulted in introducing targeted knock-ins in wheat with the frequency of 1% [42]. Furthermore, a study on *Arabidopsis* suggests that recruitment of the exonuclease with SunTag could result in 2-fold higher mutation frequency than direct fusion with the Cas9 nuclease; it also demonstrated the effectiveness of human three prime repair exonuclease 1 (TREX1) and *E. coli* exonuclease I (ExoI) in increasing the mutation frequency and deletion sizes [43].

Although increasing the expression of Cas nuclease and sgRNA, as well as the working efficiency of the system, is desirable in most cases, this may also jeopardize the final results by introducing more off-target edits. Since Cas9 can recognize sequences with slight differences to its target, the complex may bind and induce mutations in undesired sites [44]. To address this issue, numerous modified versions of the protein have been developed, producing high-fidelity variants [45]. Bioinformatics tools can be used to identify possible off-target sites in advance, as well as design highly specific sgRNA sequences, but this may not precisely predict the actual results [46]. While sequence-specific properties of the targeted site, such as melting temperature, are crucial to predict sgRNA binding, it has also been noted that the CRISPR-Cas system efficiency is influenced by expression-based characteristics, possibly due to the presence of some endogenous factors or the increased physical accessibility of the highly expressed sites in the genome [47]. Apart from using highly specific sgRNAs, alternative Cas variants, virus-based transformation methods, and adjusting Cas/sgRNA concentrations may also be of use to mitigate the off-target editing [46,48]. It has been noted, however, that integration of Cas/sgRNA in the plant’s genome could be advantageous in species that are characterized by low editing efficiency, as the continuous activity of the protein could induce new mutations in the next generations, allowing for obtaining homozygous mutants, and with highly specific gRNA design, this approach will not generate off-target edits [19,49].

## 5. Navigating Efficiency Assessments

**Figure 1:**
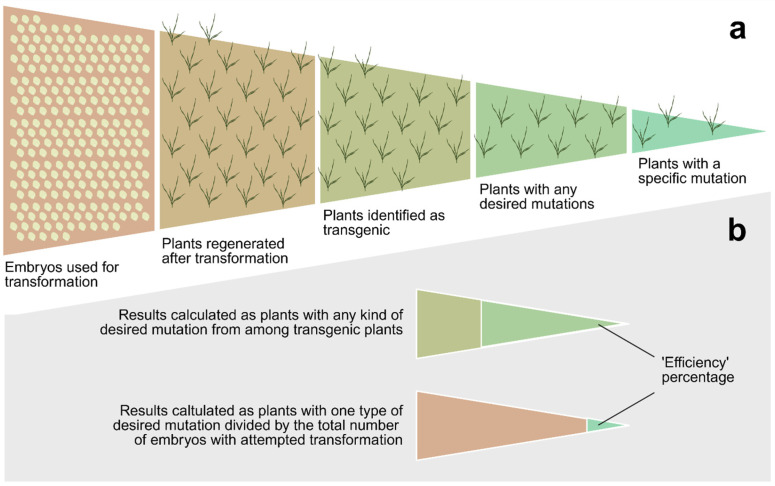
Schematic explanation of how different approaches to determining editing efficiency result in major discrepancies in the results between studies. (**a**) Steps of plant genetic transformation. (**b**) Illustration of the disproportion between the results obtained with different calculating methods. Created in BioRender. Samoń, M. (2026) https://BioRender.com/713iq1g. Accessed on 26 June 2026.

The first study reporting the application of the CRISPR-Cas technique in wheat was editing the *TaMLO* gene in protoplasts with an efficiency of 28.5% [50]. Since then, CRISPR-Cas systems have repeatedly proven to be valuable tools for versatile implementations in wheat genome editing. The modified sequences include genes affecting the yield and its quality, end-use characteristics, herbicide and pathogen resistance, and abiotic factors responses [51]. Nevertheless, since there are no universal parameters for the assessment of CRISPR-Cas system applications and the scopes of individual studies vary, it is impossible to directly compare the CRISPR techniques outside of a design of a given experiment or to outline a universal protocol of modifying the wheat genome using the CRISPR-Cas method, especially considering the diversity of possible implementations. Whereas some works offer general guidelines [44,48,52], searching through articles for reliable indications can be time-consuming and not always lead to a definitive conclusion (Figure 1). For example, the approach adopted in some studies defines the editing efficiency as the percentage of plants with an editing event from among the plants regenerated after transformation or identified as transgenic [34,36,53], whereas in other cases, the efficiency is stated in relation to the number of transformed embryos [28]—which, unlike the forementioned method, is applicable in transient expression systems as well [54,55]. Sometimes both parameters are provided, presenting the most impartial results and demonstrating significant differences between the two values, which could vary even 15-fold [30]. On the other hand, in certain works, the efficiency is not specified directly and can only be obtained by calculating numbers presented in the paper [35,56]. Moreover, in multiplex editing studies, it is vital to specify whether given values refer to the summarized mutation frequency or the frequency regarding one particular *locus* [34]. The issue is further complicated in studies that use one gRNA to target multiple *loci*, as well as in works that assess editing of homologous genes in polyploid organisms [49]. Another design parameter affecting the final efficiency is the transformed tissue—usually immature wheat embryos are used for stable transformations, whereas transient expression is studied in protoplasts [10,31]. The protoplast assays are also often used for validating methods prior to transforming the plants [44,57]. Concluding, while comparing values related to gene editing efficiency, the differences in study setups need to be taken into account, as failing to do this results in enormous discrepancies between results obtained in apparently similar experiments [6].

## 6. Base Editing

**Figure 2:**
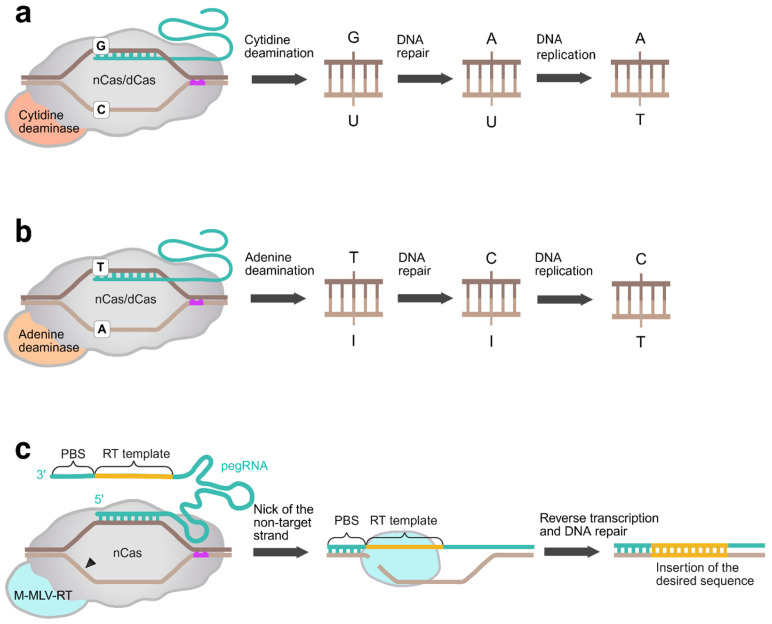
Mechanisms of Cas-based genome editing techniques. (**a**) Cytidine base editing. (**b**) Adenine base editing. (**c**) Prime editing. Brown lines—target DNA; verdigris lines—gRNA; purple segment—PAM sequence; black triangle—cleavage site; G—guanine; U—uracil; A—adenine; T—thymine; C—cytidine; I—inosine; PBS—primer binding site; RT—reverse transcriptase; M-MLV-RT—Moloney murine leukemia virus reverse transcriptase. Created in BioRender. Samoń, M. (2026) https://BioRender.com/f8a1118. Accessed on 26 June 2026.

Base editing is a derivative of the CRISPR-Cas method, which uses a deaminase domain fused with a modified Cas protein—either a nickase (nCas) or a completely inactivated nuclease (dCas)—to introduce precise substitutions without inducing double-strand breaks. The modified Cas, in conjunction with a specific gRNA particle, functions as a navigation platform, enabling the construct to bind to a specific site in a genome. Cytosine deaminase catalyzes the transition of cytosine into thymine, whereas adenine deaminase catalyzes the transition of adenine into guanine (Figure 2a,b) [58]. Both types of Cas base editors have been applied in wheat [59,60,61]. Some modifications of the technique include dual base editors, transversion base editors, and PAMless base editors [62], but to our knowledge, no applications of these methods in wheat have been reported to date.

Zong et al. tested plant base editors consisting of rat apolipoprotein B editing complex 1 (APOBEC1), cytidine deaminase, inactivated Cas9 (nCas9 or dCas9), and uracil glycosylase inhibitor (UGI), flanked with two NLSs, and verified their editing capability using the BFP-to-GFP reporter system in wheat protoplasts [61]. The fusion protein with dCas9 and nCas9 resulted in 0.3% and 6.8% of edited cells, respectively. The latter construct was subsequently used to edit the endogenous wheat *TaLOX2* gene; out of 160 immature embryos used for transformation, two plants were confirmed to be heterozygous mutants. The same plant base editor was used to target a specific position in the wheat TaALS-P174 site, and the mutations could be detected in 2.5% of the T0 plants derived from the bombarded embryos [60]. It should be noted, however, that the modifications occurred in five subsequent cytosines in the target sequence and apart from expected C to T conversions, C to G transversions were also reported; this approach could, therefore, be considered as not precise enough in some applications. The study further modified the conditions by delivering the base editor and sgRNA sequences as *in vitro* transcribed mRNAs or using two sgRNAs in simultaneous editing; the obtained mutation frequencies were 0.5% and 2.25%, respectively. Li et al. used a plant adenine base editor to edit *TaDEP1*, *TaEPSPS*, and *TaGW2* genes in wheat protoplasts, reporting editing efficiency of up to 7.5%, as well as no evidence of unintended modifications [59]. The study also verified the performance of the system in producing wheat plants with mutated *TaDEP1* and *TaGW2* genes. Out of the total number of embryos that underwent the transformation, 1.09% and 0.43% had a heterozygous mutation in either *TaDEP1* or *TaGW2*, respectively, and in five out of the seven mutant plants, the transgene vectors were not identified.

## 7. Prime Editing

Similar to base editing, prime editing is a CRISPR-Cas modification that utilizes catalytically inactive Cas as a navigation platform for an enzymatic domain. In this system, the prime editors—composed of nCas fused with Moloney murine leukemia virus reverse transcriptase (M-MLV-RT)—and prime editing guide RNAs (pegRNAs) are used to incorporate precise substitutions (Figure 2c). The pegRNA consists of sgRNA, as well as a sequence encoding the desired edit. When the prime editor binds to the selected site in the genome, nCas cleaves the non-target DNA strand, and the loose 3′ end acts as a primer for reverse transcription with the pegRNA as the template; the newly synthesized fragment is subsequently incorporated in the genome following DNA repair [63].

The original prime editing system (PE1) has been gradually improved by introducing numerous modifications. In PE2, employing an engineered RT enhances the efficiency of the editor [64]. PE3 and PE3b systems include additional sgRNA sequences to nick the non-target or target strand, respectively [65]. Additionally, using engineered pegRNAs (epegRNAs), which include structured RNA motifs, was shown to improve the performance of prime editors [66]. Transient inhibition of DNA mismatch repair by the co-expression of MLH1dn protein also increases prime editing efficiency and reduces rates of undesired indels. Adoption of this strategy in the PE2 and PE3 systems resulted in the development of the PE4 and PE5 editors, respectively [67]. These improvements were further complemented by adjusting the architecture of the prime editor in PEmax systems [67]. PE6 editors incorporate more compact reverse transcriptases, whereas in PE7, fusing the N-terminal fragment of La protein to PEmax increases pegRNA stability [68,69]. Recently developed PE8 systems employ computationally redesigned reverse transcriptases with improved stability and expression levels [70].

TwinPE uses two pegRNAs targeting opposite DNA strands to generate larger insertions, sequence replacements, and deletions. Combining this technique with a site-specific integrase also allowed for integration and inversions of multi-kilobase DNA fragments [71]. In this strategy, prime editing is used to install attachment sites of site-specific integrases in the desired genomic *loci*; the inserted sequences are subsequently replaced with desired DNA cargos by corresponding integrases. Methods adopting this approach include programmable addition via site-specific targeting elements (PASTE) and prime editing-assisted site-specific integrase gene editing (PASSIGE), which were demonstrated to carry out targeted insertions of large sequences in mammalian cells, as well as Prime editing-mediated Recombination Of Opportune Targets (PrimeRoot), a plant-optimized integrase-based editor [72,73,74].

Since this technique was developed recently, there are not many examples of its implementation in wheat genetic modifications. Lin et al. reported single-nucleotide substitutions with frequencies of up to 1.4% after prime editing of *TaUbi10*, *TaGW2*, *TaGASR7*, *TaLOX2*, *TaMLO*, and *TaDME1* genes in wheat protoplasts using two variants of plant prime editing systems (PPEs): PPE3 and PPE3b [75]. Ni et al. tested various pegRNA modifications, prime editor modifications, and multiplex pegRNA architectures to develop an efficient prime editing system for wheat [34]. It was demonstrated that adding the tevopreQ1 motif to the 3′ end of pegRNA, as well as incorporating mutated proteins and optimized nuclear localization signals in the prime editor, termed ePPEplus, can significantly increase editing frequencies without the penalty of undesired editing byproducts. Moreover, the Csy4 processing system and CmYLCV promoter proved to be the most efficient for pegRNA expression in the case of multiplex editing compared to the tRNA system, ribozyme system, and monocistronic constructs with RNA polymerase III promoters. Using these components allowed for simultaneous editing of 4–10 targets in wheat protoplasts with an average efficiency of 7.4–10.3%; it should be noted, however, that these values varied significantly between individual genes. In transgenic wheat plants, multiplex targeting of eight genes resulted in overall editing efficiency (i.e., the percentage of plants with a mutation in at least one target gene divided by the total number of plants regenerated after transformation) of 94.1%, and the mutagenesis in individual sites was between 19.6% and 86.3%.

The DualPE method combines the ePPEplus editor with paired epegRNAs to introduce insertions, deletions, and invertions, similar to the twinPE technique [76]. The application of DualPE in wheat protoplasts generated targeted deletions of up to 365.9 kb, with frequencies ranging between 0.1% and 30.9% and an editing accuracy of 83.9–99.1%. In transgenic wheat plants, precise deletions of 365.9 kb and 2 Mb chromosomal fragments were achieved with frequencies of 10.5% and 7.7%, respectively; in both cases, no off-target mutations were detected. The efficiency of using DualPE in wheat protoplasts for sequence replacement ranged from 0.4% (replacement of 258 kb with 34 bp) to 43% (replacement of 41 bp with 50 bp), with an average accuracy of 93.5%. Targeted replacement of 567 bp with 157 bp in transgenic wheat resulted in 28.6% of mutant plants, half of which contained the desired edit. The method was also demonstrated to produce targeted inversions in wheat with frequencies of 0.2–8.4% in protoplasts and 21.9–51.5% in transgenic plants. Notably, although the reported efficiency rate for inversion of a 252.6 kb fragment in protoplasts was 0.2%, it reached 27.6% in the case of a 205.4 kb sequence in wheat plants, with all of the mutants identified as precisely inverted [76].

TwinPE-based gene knockout (TKO) is a recently developed approach for inserting stop codon clusters into plant genomes [77]. The technique was demonstrated to outperform classic Cas9-based systems in wheat protoplasts, generating knockouts of individual homologs in eight genomic targets, with an average efficiency of 65.9% and low rates of 3n indels. The performance of the TKO editor was also evaluated in wheat plants, achieving triple knockout of the *TaKRN2* gene in 1.9% of plants regenerated after particle bombardment.

The programmable chromosome engineering (PCE) system employs a prime editor, a modified Cre recombinase, and improved Lox sites to facilitate large-scale genomic edits spanning up to megabases. The method was reported to outperform the PrimeRoot editor in wheat protoplasts, allowing for insertions of 720 bp, 2.4 kb, and 18.8 kb DNA fragments with a frequency of up to ~8%. The application of PCE to insert a 786 bp fragment at two genomic sites in wheat resulted in precise integration in 9.7% and 5.4% of regenerated plants [78].

## 8. Epigenetic Editing

Employing catalytically inactive Cas proteins as navigation platforms for effector domains further expands the usability of CRISPR-based methods, allowing for applications other than altering the genomic nucleotide sequence. First, binding of the Cas enzyme may simply physically block transcription activation or elongation in the target genomic *locus* [79]. Additionally, CRISPR activation (CRISPRa) and CRISPR interference (CRISPRi) systems have been engineered by fusing the C-terminus of dCas9 with transcriptional activation domains (TADs) or transcriptional repression domains (TRDs), respectively [80]. Zhou et al. have applied the CRISPRa/CRISPRi system to guide transcriptional activator (6 × TAL-VP128) or transcriptional repressor (3 × SRDX) to the promoter of the *PDS* gene in wheat, demonstrating the effectiveness of the constructs in up- or down-regulating the transcription, as well as the inheritance of the effect [81]. Furthermore, Xu et al. have shown that DLN144, DLS, and MIX are other TRDs that are suitable for CRISPRi in wheat [82]. Fusing the inactivated Cas with enzymes altering DNA methylation or histone methylation/acetylation allows for manipulation of epigenetic modifications [83,84]. Gene expression at the post-transcriptional level may also be regulated by targeting RNA molecules, either transcripts or non-coding RNAs. Whereas Cas-based techniques can be employed for editing or identifying the genomic *loci* of non-coding RNAs (ncRNAs), using the CRISPR system consisting of a nuclease targeting single-stranded RNA could be of use to directly target coding or regulatory RNAs [84,85]. Although those techniques have been applied in some plant species, no reports of their use in wheat could be found.

## 9. Different Cas Variants Applied in Wheat

**Figure 3:**
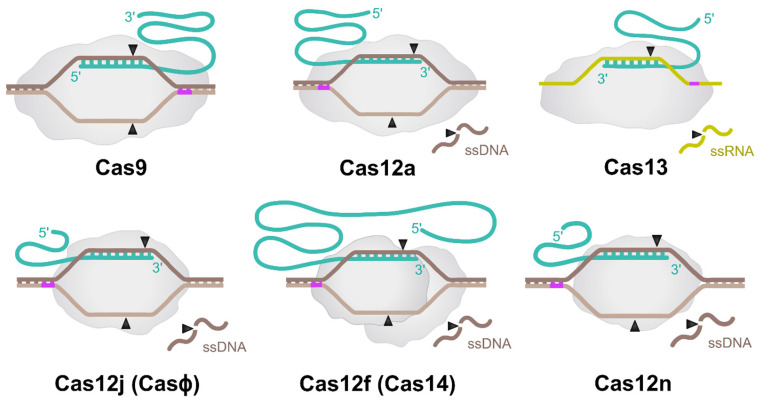
Comparison of selected Cas nucleases. Brown lines—target DNA; lime lines—target RNA; verdigris lines—gRNA; purple segment—PAM sequence; black triangles—cleavage sites. Created in BioRender. Samoń, M. (2026) https://BioRender.com/1g61b1f. Accessed on 26 June 2026.

Apart from *Staphylococcus pyogenes* Cas9, which is the most widely employed nuclease in the CRISPR method, other Cas nucleases with different characteristics have also been used in the technique, significantly expanding the available toolkit and possible applications (Table 1, Figure 3). For example, Zhong et al. have developed a genome editing system using Cas9 from *Lactobacillus rhamnosus* (LrCas9), a nuclease recognizing NGAAA PAM, more tolerant to lower temperatures than the more commonly used variants [86]. The study demonstrated the effectiveness of LrCas9 in many plant species, including wheat. The protein was also reported to be more effective than SpCas9-NG, SpRY, and LbCas12a, as well as being suitable for facilitating multiplex editing, generating stable mutations, and engineering base editors or CRISPRa/CRISPRi systems. Cas12a (previously known as Cpf1), a nuclease recognizing T-rich PAMs, is considered to be more specific than Cas9, possibly because it requires a longer spacer; however, successful editing could also be achieved with shorter spacers [87,88,89,90]. Cas12a forms complexes with short crRNA molecules (~42 nt) and generates staggered-ended cuts upstream of the PAM sequence; additionally, this protein is capable of cleaving RNA, allowing for processing a multi-crRNA array [88,91]. Cas12a has been used in wheat to edit the *TaPDS* gene, resulting in higher editing efficiency and fewer off-target mutations when compared to Cas9 [92]. Wang et al. used native and engineered variants of Cas12a to target wheat *TaAn-1*, *TaGASR7*, *TaGS3*, *TaGSE5*, *TaGW2*, *TaGW7*, *TaPDS*, and *TaSPL16* genes; it was also proven that higher temperatures and flanking the crRNAs with ribozymes improve the system’s efficiency [14]. Increasing the temperature may not be optimal for the plants, however; this issue was addressed by engineering temperature-tolerant LbCas12a variants [93]. Optimized base editing of *TaLOX2* and *TaMLO* genes with a system composed of Cas12a has resulted in 30–50% of the transformed wheat plants having at least one targeted edit, and the mutations were highly inheritable [94]. Notably, this study addressed some factors affecting the performance of the system in plants, i.e., type and position of NLS, terminator, and crRNA architecture, demonstrating the efficiency of the construct with two bipartite (BP) NLSs, G7 terminator, mutation (D156R) in the LbCas12a protein, crRNA containing two mature direct repeats (DRs), TaU3 promoter, polyT signal, and truncated tRNA. MAD7, a Cas12a-like nuclease recognizing ATTN PAMs, has been used to edit *TaDEP1* and *TaDME-T1* genes in wheat protoplasts and performed better than or similar to LbCas12a, with an editing efficiency of up to 38.7%. Moreover, this nuclease was proven to be highly specific, as well as capable of multiplex gene editing and—upon modification—recognizing alternative PAMs or being used in the APOBEC-Cas fusion-induced deletion (AFID) system. Targeting *TaDEP1* and *TaVRN1* genes by CRISPR-MAD7 in transgenic plants resulted in mutagenesis of 1.5% and 3.0%, respectively, calculated as the percentage of mutants divided by the number of wheat embryos used for transformation [95]. Another nuclease implemented in the CRISPR editing technique is CasΦ2, a protein derived from huge bacteriophages. Due to its small size (700–800 aa), T-rich PAM association, high specificity, and wide range of working temperatures, CasΦ2 is considered to be an attractive new tool for genome editing. Similar to Cas12a, it generates staggered ends and has ribonuclease activity; therefore, it is able to process pre-crRNA molecules [96]. Testing the codon-optimized and engineered variants of the protein in wheat protoplasts demonstrated that VCasΦ2, adjusted by deleting amino acids of the native protein and inserting the GSSG sequence, was the most effective system for gene editing, with an efficiency of 13–41%. The method was further improved by optimization of nuclear localization signals and using paired crRNAs. Following its inactivation, the protein was used to create functional adenine and cytosine base editors and was confirmed as highly specific, allowing for editing *TaGW2*, *TaALS*, or *TaNAC2* genes and resulting in editing efficiency of up to approximately 4%. In transgenic plants, the cytosine base editor induced desired conversions in the *TaGW2* gene and the *TaPIN* gene with the frequency of 9.1% and 6.9%, respectively, whereas in the case of the adenine base editor used for editing the *TaALS* gene, the editing frequency was 6% [97]. A recent study investigating the performance of Cas12i3, a relatively compact nuclease recognizing TTN PAM, demonstrated the functionality of this protein in transgenic wheat [98]. The highest editing rates were achieved by employing Cas12i3-5M, a variant with effectiveness-enhancing mutations, fused with T5 exonuclease, as well as applying optimized gRNA expression architecture, i.e., composite 35S-CmYLCV-U6 promoter and flanking the sequence with tRNA and hepatitis delta virus (HDV) ribozymes. Using this system in stable wheat lines to edit *TaARE1*, *TaHRC*, *TaSBEIIa*, and *TaPsIPK1* genes resulted in 60.95–88.99% of the transgenic lines carrying an independent mutation. The study also reported that co-expressing T5E exonuclease increased the frequency and size of induced deletions.

## 10. Delivery Systems and Transformation Methods

The components of the CRISPR system are usually delivered to plant cells as DNA sequences. Protoplast transfection is typically used for transient expression, whereas biolistic methods or *Agrobacterium*-mediated transformation are utilized to obtain stable expression in plants [10]. Integration of the foreign DNA in the plant genome may have some side effects, however, like disruption of a crucial genomic site or increased off-target mutation rate due to prolonged expression of the CRISPR machinery; it is also more problematic legislation-wise and potentially hazardous to the environment [10,129,130]. Zhang et al. established a protocol of transient transgene-free expression of CRISPR-Cas elements in wheat [131]. In this technique, plasmids expressing Cas9 and sgRNA were delivered via particle bombardment to immature wheat embryos. The method was used to produce plants with homozygous mutations, which could be transferred to the progeny, with a low level of undesired editing and a high number of transgene-free mutants; it also allowed for shortening the regeneration period, as the plants were grown without any herbicide selection. Moreover, the system was utilized to deliver *in vitro* transcripts instead of DNA particles. Although the mutagenesis rate was lower, all of the obtained mutants were reported as transgene-free. Another form of DNA-free delivery is ribonucleoprotein complexes (RNPs), composed of proteins and RNA. They were shown to be efficient in inducing mutations in immature wheat embryos, providing reduced frequency of off-target mutations, as well as transgene-free plants [55]. Biolistic delivery of RNPs was improved by Tanaka et al., who reported that using 0.6 μm gold particles and 24 h incubation at 34 °C increases the editing efficiency [30]. Although biolistic methods are more customizable regarding the type of cargo (DNA, RNA, or RNP), size, and amount of gold particles or bombardment parameters, they may generate multiple copy insertions and be related to lower regeneration efficiency [30,132]. On the other hand, *Agrobacterium*-based transformation typically results in a single-copy insertion, but the technique is considered to be ineffective for some species, genotypes, and tissues [30,132]. These systems can also be used for the transient expression of the CRISPR-Cas system in plants to generate transgene-free mutants [133]. Interestingly, studies on dicotyledon species have shown that *Agrobacterium*-mediated transformation can be significantly improved with a ternary vector system for overexpression of the bacterial virulence genes and degradation of plant defense signals [134]. Using noninfectious geminivirus-based DNA replicons to provide a high copy number of the effector nuclease, as well as the guide RNA sequences, may additionally increase gene targeting frequencies; in wheat, a replicon based on wheat dwarf virus was shown to facilitate both targeted deletions and knock-ins, as well as being suitable for multiplex gene targeting [26,135].

As an alternative to the commonly employed techniques, Doyle et al. demonstrated that carbon dots carrying a plasmid with Cas9 and gRNA can be a suitable delivery agent for the CRISPR-Cas system; although the scope of the study included only transient transformation by applying spray to mature wheat plants leaves, it was described as easily applicable, non-toxic, not impairing to the plants’ physiological processes, and having potential to be used for creating stable edits by targeting the germline cells [136]. Bhowmik et al. have reported efficient CRISPR-Cas editing in wheat microspores after transformation via electroporation, as this method is relatively simple and effective [137]. Using microspores as transformed material was noted to be relatively less laborious and time-consuming. Moreover, in haploid cells, there are fewer alleles to be edited, and microspores could be used to obtain homozygous double haploid mutant plants. As demonstrated by Budhagatapalli et al., double haploids can also be achieved by intergenic crossing [138]. In this experiment, wheat was pollinated with pollen of transgenic maize expressing Cas9 and wheat-specific gRNA. Subsequent elimination of maize chromosomes and colchicine treatment of haploid plants enabled obtaining transgene-free wheat plants with desired mutations, which were frequently homozygous. Hamada et al. demonstrated that successful transient transformation and CRISPR-Cas editing can be achieved by *in planta* bombardment of shoot apical meristems (SAMs) of imbibed wheat seeds, claiming the method to be suitable for DNA-free transformation of recalcitrant cultivars [54]. In this study, out of the total number of bombarded embryos, 5.2% were later identified as mutant T0 plants, 1.4% were determined to carry a mutation in T1, and the transgene was not detectable in any of the T1 specimens. The method of *in planta* SAM bombardment was later confirmed by Liu et al. to be capable of inducing targeted mutations in elite wheat cultivars, which could be problematic in tissue culture [139].

Recently developed virus-induced gene editing (VIGE) is another system, utilizing virus-based vectors to deliver CRISPR-Cas machinery components to plant cells. The advantages of this technique include an increased number of RNA copies, high levels of gene expression, the capability of targeting multiple targets, and a shortened transformation/regeneration period, or bypassing this step of plant transformation [140,141,142,143]. Employing virus-based systems also prevents stable integration of the foreign DNA in the plant genome and minimizes off-target effects [144]. However, adoption of the method has remained challenging due to the inherent limitations of viral vectors, i.e., host range, engineerability, systemic spread, and cargo capacity. Applications of the VIGE technique have focused mainly on delivering gRNA to plants with stable overexpression of Cas nucleases, followed by subsequent crossing of the edited specimens with the wild-type plants to segregate out the *Cas* gene and generate non-transgenic plants with desired mutations [134,143]. This method, employing barley stripe mosaic virus to deliver the sgRNA sequences, was demonstrated to be applicable in wheat by targeting the *TaGASR7* gene [142]. The technique was confirmed and further explored by Li et al. [145]. Using the system to edit *TaGASR7*, *TaGW2*, and *TaPDS* genes in three transgenic wheat varieties was reported as a highly efficient method to obtain heritable mutations and generate transgene-free progeny via crossing with wild-type plants; it was also effectively applied in multiplex editing. Notably, a recent study reported using an RNA vector based on barley yellow striate mosaic virus (BYSMV) to express both Cas9 and sgRNA in wheat [146]. To further improve the performance of the system, both of the delivered sequences were fused with a transfer RNA-like structure (TLS). Deriving progeny from infection-induced tillers allowed for obtaining transgene-free plants with inherited bi-allelic or homozygous mutations, resulting in the development of the virus-induced genome editing in tillers (ViGET) method, which was also demonstrated to be applicable in some commercial wheat varieties [146].

## 11. Tools and Methods of Potential Application

The CRISPR-Cas technique and related genome editing methods are still being developed to facilitate different activities of the Cas proteins or to enhance the performance of the existing systems. Although many of the available tools have been implemented in modifying the wheat genome, some of them still await being applied to this species. For example, no reports of using the engineered SpCas9 variants with different PAM association or enhanced specificity could be found during the research for this review [4,44]. Similarly, Cas13, a nuclease targeting single-stranded RNA, as well as recently developed Cas12 systems, like highly precise Cas12b or very compact Cas12f, have yet to be used in wheat [9,112,147,148]. Due to the unique characteristics of these tools, their potential employment seems to be worth investigating (Table 1 and Table 2).

Although type I CRISPR-Cas systems are not commonly used for genetic engineering, they may be useful in plant modifications, as they have been proven to be capable of generating long deletions with low off-target effects [162]. Additionally, the use of ‘typical’ Cas proteins for generating paired DSBs resulting in targeted deletion or inversion of a large chromosome fragment, albeit reported in other plants, has not been performed in wheat [163]. As structural chromosome variations have been associated with agronomic traits of this species, such manipulations could be useful in generating new lines; this approach was also noted to potentially produce new germplasm by eliminating native chromosomes or generating new artificial chromosomes [51,164]. A presumably applicable CRISPR system was developed by Liu et al. and tested in rice, maize, and tomato [36]. The system is based on Mb3Cas12a, a Cas12a ortholog from *Moraxella bovoculi*, which recognizes TTV PAM. Further optimization of the method led to engineering a highly precise, single transcription unit with enhanced working efficiency at lower temperatures, corresponding to conditions of plant cultivation. Novel technologies based on Cas12k, a protein associated with Tn7-like transposons, or CRISPR-associated transposases (CAST), were demonstrated to generate targeted insertions [165,166]. Notably, Liu et al. developed a transposase-assisted target site-integration system (TATSI) for targeted integration in plants using Cas9/Cas12a nucleases and rice Pong transposase [167]. The technique was successfully deployed in *Arabidopsis thaliana* and soybean (*Glycine max*) to introduce sequence-specific insertions of up to nearly 9 kb. Although the authors indicated certain shortcomings, they deemed the method potentially usable in nearly every transformable crop plant; to our knowledge, however, no subsequent application of this system has been reported to date. Another recently established modification of the CRISPR system involves delivery of the system components by transporting from a transgenic graft. As presented by Yang et al. in *Arabidopsis thaliana* and *Brassica rapa*, transcripts of Cas9 and sgRNA fused with the tRNA-like sequence (TLS), expressed in transgenic rootstock, can be translated and induce edits in wild-type scions, possibly enabling the obtaining of edited and transgene-free seeds without the need for back-crossing, significantly accelerating the production of gene-edited plants. Although not demonstrated, the technique was predicted to be applicable in monocotyledonous species [168].

There are also many adaptations of the CRISPR genome editing technique, which were demonstrated to be useful in certain setups but have yet to be deployed in plants. For example, studies show that chemical modifications of gRNA and specific arrangements of its architecture, like incorporating a hairpin structure or extending the termini, can enhance editing efficiency [169,170,171]. Similarly, modifications of Cas protein can alter the performance of the CRISPR system, as presented with the aforementioned examples of fusing Cas with NLS or an exonuclease. Interestingly, it was observed that a chimeric protein of Cas9 and GFP had higher editing efficiency than the native Cas9, which was hypothesized to be explained by the possible favorable conformational arrangement of Cas9 in the chimeric protein [172]. It was also shown that fusing Cas12 with crRNA and chemical modifications of Cas9 can enhance efficiency or facilitate new functions of the system [173,174]. A novel approach to expand the editing window of targeted mutagenesis uses a programmable nuclease coupled with an error-prone DNA polymerase [175,176]. Alternatively, a recently developed method of click editing, which employs a high-fidelity DNA-dependent polymerase and DNA template, was demonstrated to produce precise modifications of different types [177]. These techniques have not been implemented in plants to date; considering their biotechnological potential, they could be powerful tools in the genomic engineering of crop species.

## 12. Emerging Innovations: Miniature Nucleases, Viral Delivery, and Transposons and Ribozymes

CRISPR systems are commonly found in genomes of prokaryotic organisms, and some Cas proteins, like CasΦ2 or Casλ, were discovered in bacteriophages [122,178,179]. These natural reservoirs can, therefore, be a source of new genome editors of facilitative characteristics, complementing the ongoing efforts to adjust the tools that are already in use. Among the recently characterized Cas proteins, there are many variants defined as ‘compact’ or ‘miniature’ compared to Cas9 and Cas12a (~1200–1400 aa), such as *Actinomadura craniellae* Cas12n (506 aa), *Acidibacillus sulfuroxidans* Cas12f (422 aa), *Syntrophomonas palmitatica* Cas12f (497 aa), or *Armatimonadetes bacterium* Cas12l (Casπ) (867 aa) [128,180,181,182]. Additionally, the discovery of OMEGA (obligate mobile element guided activity) nucleases TnpB and IscB (~400 aa), which are transposon-encoded, RNA-guided proteins hypothesized to be the ancestors of Cas12 and Cas9 nucleases, respectively, allowed for the development of particularly small genome editing systems [183,184,185,186]. Eukaryotic RNA-guided nucleases, termed Fanzor, were also recently characterized and assessed in the context of biotechnological applicability [187]. Although the miniature nucleases are typically outperformed by Cas9- or Cas12-based editors when directly compared, their limitations could be compensated by tinkering with the enzymes’ properties and delivery methods, in particular using viral vectors for expressing the proteins in the plant host [119,128,147,188,189,190]. A noteworthy strategy to handle the cargo size restrictions is loading the viral vectors with fragments of the transgene and reassembling the functional unit in the target host cells. For example, Chen et al. performed DNA-free gene editing in cotton using a four-component barley stripe mosaic virus (BSMV) system for the expression of sgRNA and split Cas9 transcript sequence [191]. The same vector was used by Hu et al. to deliver intein-mediated split SaCas9 for VIGE in sheepgrass [144]. Although the reported editing efficiencies were relatively high, it was noted that large inserts may impair pathogenicity or mobility of the virus since no edits could be found in leaves that developed after the infection, indicating the necessity of further adjustment of viral vectors or reducing the cargo size. However, adapting the most compact OMEGA and Fanzor nucleases as genome editors may be challenging, considering their hypothesized function in propagation of mobile genetic elements and consequent low activity and/or strict regulation [187]. This issue may also hinder the performance of other cutting-edge transposon-associated techniques, such as INTEGRATE or bridge RNAs, as well as the prospect of adapting the recently characterized sequence-specific nucleolytic ribozymes of class II introns as compact protein-free genome editing tools [192,193,194].

## 13. Pending Issues

As confirmed by numerous examples, CRISPR-Cas technology is a valuable and versatile tool for genome editing. Adaptation of the systems that consist of the newly identified Cas proteins and the engineering of the ones with already established applications suggest that the potential of this method is still not fully exploited. Nevertheless, there are pending challenges that need to be overcome—or mitigated—before these genome editing approaches become reliable tools for routine crop improvement. Despite over a decade of advances in adapting the CRISPR-Cas systems for modifying the wheat genome or its expression, efficient editing is still associated with technical difficulties regarding delivery, transformation, expression, efficiency, specificity, and transgene integration [195], and even though the technique was verified to be applicable in research setups, its widespread use in agriculture and food production still seems to be a distant prospect. One of the major technical challenges is applying the technique in elite crop varieties, which tend to be recalcitrant to genetic transformation [17,196]. Although this issue can be addressed by improving the transformation protocol, for example, incorporating morphogenic regulators or adapting virus-based methods to bypass the tissue culture stage, more efficient and versatile delivery methods for wheat are needed [51,197]. Other concerns regard the presence of potential off-target effects, which could result in undesired characteristics in the edited organism [198]. To mitigate this problem, it is necessary to properly design the sgRNA sequences and evaluate the possibility of off-target binding in the genome of interest; also, adapting a system of transient expression for inducing genome modifications, as well as employing a high-fidelity Cas nuclease or a paired nickase, could be useful. It should be noted, however, that the Cas variants of enhanced specificity may also have lower editing efficiency [44,199,200], and a trade-off trend between activity and specificity has been observed [201].

A significant factor that further prevents the application of CRISPR-Cas technology in certain regions is the legal status of the organisms obtained with this method. Whereas many areas have not defined the status of the genome-edited (GE) organisms, the established regulations vary remarkably between different regions. According to the process-based approach implemented in regions like the European Union, the genome-edited organisms are considered GMOs and, therefore, must conform to the required norms and procedures, i.e., complex, long-term evaluation, which is affordable only by the biggest companies in the breeding industry [202]. It has been noted that this approach is not justified by science-based arguments, as the final product of genome editing is not distinguishable from an organism that obtained a mutation without any intentional intervention; moreover, the modern genome editing techniques are far more precise than the classical mutagenesis, which usually generates numerous off-site mutations [202,203,204]. On the contrary, in countries like Canada, the United States, Japan, Argentina, or Australia, the edited organisms are treated much less strictly, with the successful commercialization of some GE plants [205,206,207]. This approach correlates with the consumers’ attitude towards the edited crops [203]. Although it was noted that the public opinion is affected by the regulatory framework, i.e., strict policy may enhance the general concerns, the relationship is in fact mutual, and the legislation is influenced by the people’s views. A survey study conducted among a panel of experts by Lassoued et al. has revealed that political involvement in the regulatory process is considered to be the most limiting factor for the development of new breeding techniques, including gene editing [208]. This observation corresponds with the findings of de Lange et al., which show that, according to the plant scientists, the consumer perceptions/knowledge gap and policy/legal issues are the main barriers to CRISPR technology adoption [209]. On the other hand, however, increased control of the genome-edited organisms in the initial stage may help to get society familiarized with the notion and to better troubleshoot any potential setbacks that could result in public controversies, jeopardizing the general approval of these techniques. Interestingly, although genome editing is a relatively new concept, gene-edited crops seem to be generally more accepted than genetically modified organisms [210,211,212]. Nevertheless, public opinion is still one of the many unresolved issues associated with the technology, which needs to be addressed considering the increasing urgency of developing crops with improved traits [213]. To further ensure effective utilization of the genome editing tools in the face of real-life challenges, it is necessary to properly identify the impact of the desired changes on the modified organism. According to the predictions, crop plants are going to be exposed to a growing number of stresses, often occurring simultaneously; it is, therefore, desirable to develop lines resilient to numerous different factors [214]. However, it was observed that the plant response to a combination of multiple stresses is often unique and impossible to predict based on the responses to each of the individual factors, and it should thus be treated as a new type of stress [215,216]. The role of fundamental research in investigating the effect of the actual field environment on plants’ physiological processes is, therefore, crucial for the identification of underlying mechanisms, as well as cross-talks between different pathways, and the capability of correctly predicting results of intended interferences.

## Figures and Tables

**Table 1:** Selected examples of Cas proteins employed in the CRISPR-Cas genome editing method.

Name	Organism of Origin	Classification	Size	PAM Sequence (5′-3′)	PAM in Relation to the Protospacer	Cut Site in Relation to the PAM	Ends	Guide RNA Structure	Protospacer Length	Targeted Cleavage Substrate; Additional Activity	References
SpCas9	*Streptococcus pyogenes*	Class 2, type II	1368 aa	NGG, NGA, NAG	Downstream	Upstream	Blunt	crRNA + tracrRNA/sgRNA	20 nt	dsDNA	[11,99,100,101]
SaCas9	*Staphylococcus aureus*	Class 2, type II	1053 aa	NNGRRT	Downstream	Upstream	Blunt	crRNA + tracrRNA/sgRNA	20 nt	dsDNA	[101,102,103]
ScCas9	*Streptococcus canis*	Class 2, type II	1375 aa	NNG	Downstream	Upstream	Blunt	crRNA + tracrRNA/sgRNA	20 nt	dsDNA	[104,105]
CjCas9	*Campylobacter jejuni*	Class 2, type II	984 aa	NNNVRYM	Downstream	Upstream	Blunt	crRNA + tracrRNA/sgRNA	22 nt	dsDNA	[99,101,106]
NmeCas9	*Neisseria meningitidis*	Class 2, type II	1082 aa	NNNNGATT	Downstream	Upstream	Blunt	crRNA + tracrRNA/sgRNA	24 nt	dsDNA; tracrRNA-independent cleavage of ssDNA	[107,108]
CoCas9	*Collinsella* spp.	Class 2, type II	1004 aa	NNNNGWNT, NNNNGCDT, NNNNATDT	Upstream	Upstream	Blunt	crRNA + tracrRNA/sgRNA	22–24 nt	dsDNA	[109]
FnCas12a	*Francisella novicida* U112	Class 2, type V	1300 aa	TTN	Upstream	Downstream	Staggered	crRNA	Min. 22 nt	dsDNA; crRNA processing	[36,87,88,110]
AsCas12a	*Acidaminococcus* spec. BV3L6	Class 2, type V	1307 aa	TTTV	Upstream	Downstream	Staggered	crRNA	Min. 22 nt	dsDNA; crRNA processing	[36,88,110]
LbCas12a	*Lachnospiraceae bacterium* ND2006	Class 2, type V	1228 aa	TTTV	Upstream	Downstream	Staggered	crRNA	Min. 22 nt	dsDNA; non-specific ssDNA cleavage, crRNA processing	[88,110]
Mb3Cas12a	*Moraxella bovoculi*	Class 2, type V	1261 aa	TTV	Upstream	Downstream	Staggered	crRNA	19–23 nt	dsDNA; crRNA processing	[36,88,110,111]
Cas12b (C2c1)	*Alicyclobacillus acidiphilus*	Class 2, type V	1129 aa	TTN	Upstream	Downstream	Staggered	crRNA + tracrRNA/sgRNA	20 nt	dsDNA; collateral ssDNA cleavage	[112,113,114]
LshCas13a (C2c2)	*Leptotrichia shahii*	Class 2, type VI	1389 aa	Protospacer flanking sequence (PFS) of HHH	N/A	N/A	N/A	crRNA	28 nt	ssRNA; collateral ssRNA cleavage	[88,115,116]
Lwa13a (C2c2)	*Leptotrichia wadei*	Class 2, type VI	1152 aa	None	N/A	N/A	N/A	crRNA	20 nt	ssRNA	[117]
Cas12i	Metagenomic database	Class 2, type V	1033–1093 aa	TTN	Upstream	Downstream	Staggered	crRNA	20 nt	dsDNA; collateral ssDNA cleavage	[118]
Cas12f (Cas14)	*Syntrophomonas palmitatica*	Class 2, type V	497 aa	TTC	Upstream	Downstream	Staggered	crRNA + tracrRNA/sgRNA	16–18 nt	dsDNA, ssDNA; collateral ssDNA cleavage	[119,120,121]
Cas12j (CasΦ)	Huge phages	Class 2, type V	700–800 aa	TBN	Upstream	Downstream	Staggered	crRNA	14–20 nt	dsDNA; non-specific cleavage of ssDNA, processing crRNA	[97,100,122,123]
Cas12e (CasX)	*Deltaproteobacteria bacterium*	Class 2, type V	986 aa	TTCN	Upstream	Downstream	Staggered	crRNA + tracrRNA/sgRNA	20 nt	dsDNA	[116,124]
Cas12k	*Scytonema hofmanni*	Class 2, type V	639 aa	GGTT	Upstream	N/A	N/A	crRNA + tracrRNA/sgRNA	16–20 nt	N/A	[125]
Cas12m	*Mycolicibacterium mucogenicum*	Class 2, type V	596 aa	TTN	Upstream	N/A	N/A	crRNA	20 nt	N/A	[126]
Cas12g	Hot spring metagenome	Class 2, type V	767 aa	None	N/A	N/A	N/A	crRNA + tracrRNA	24 nt	ssRNA; collateral ssDNA and ssRNA cleavage	[127]
Cas12n	*Actinomadura craniellae*	Class 2, type V	506 aa	AAN	Upstream	Downstream	Staggered	crRNA + tracrRNA/sgRNA	20 nt	dsDNA; collateral ssDNA cleavage	[128]

**Table 2:** Selected examples of engineered Cas variants.

Name	Modification	Reference
nCas9	Specific mutation (D10A or H840A) leading to inactivation of one of the nuclease domains of Cas9, resulting in nickase activity (cleavage of one DNA strand)	[149]
dCas9	Mutation in both of the Cas9 nuclease domains; the protein cannot cleave DNA, but it can bind gRNA and the target site	[149]
VQR-Cas9	Different PAM association (NGA)	[10]
EQR-Cas9	Different PAM association (NGAG)	[10]
VRER-Cas9	Different PAM association (NGCG)	[10]
SpCas9-NG	Different PAM association (NG)	[10]
xCas9	Different PAM association (NG, GAA, GTA)	[10]
SpMacCas9	Different PAM association (NAA)	[149]
iSpMacCas9	Improved SpMacCas9	[149]
XNG-Cas9	NG, GAG, GAA, and GAT PAMs	[150]
eSpCas9	High-fidelity variant	[151]
SpCas9-HF1	High-fidelity variant	[152]
HeFSpCas9s	High-fidelity variant	[153]
HypaCas9	High-fidelity variant	[154]
EvoCas9	High-fidelity variant	[155]
Sniper2L	High-fidelity variant	[156]
SuperFi-Cas9	High-fidelity variant	[157]
dCas12a	Mutation in the RuvC domain disabling the cleavage activity	[149]
enAsCas12a	Higher activity in lower temperatures	[149]
ttLbCas12a	Higher activity in lower temperatures	[149]
Cpf1-RR	Different PAM association (TYCV)	[158]
Cpf1-RVR	Different PAM association (TATV)	[158]
SpG	Different PAM association (NGN)	[159]
SpRY	Different PAM association (NRN, NYN with reduced efficiency)	[159]
ScCas9++	Higher efficiency	[160]
LbCas12a-RRV	Higher editing efficiency in plants, even at the non-canonical TTV PAMs	[161]
Mb3Cas12a-RRR	Higher activity in lower temperatures in plant cells	[36]
MAD7	Engineered nuclease based on *Eubacterium rectale* Cas12a, recognizing YTTV PAM	[95]
MAD7-RR	MAD7 with altered PAM association (TYCV)	[95]
MAD7-RVR	MAD7 with altered PAM association (TATV)	[95]
NCasΦ	CasΦ variant with enhanced cutting rate	[123]
VCasΦ	CasΦ variant with enhanced cutting rate	[123]

## Data Availability

No new data were created or analyzed in this study. Data sharing is not applicable to this article.
